# Somatic embryogenesis, tetraploidy, and variant leaf morphology in transgenic diploid strawberry (*Fragaria vesca* subspecies *vesca* ‘Hawaii 4’)

**DOI:** 10.1186/1471-2229-14-23

**Published:** 2014-01-13

**Authors:** Qian Zhang, Kevin M Folta, Thomas M Davis

**Affiliations:** 1 Department of Biological Sciences, University of New Hampshire, Durham, NH 03824, USA; 2 Horticultural Sciences Department and the Graduate Program in Plant Molecular and Cellular Biology, 1301 Fifield Hall University of Florida, Gainesville, FL 32611, USA

**Keywords:** *Fragaria*, Strawberry, Transgenes, Leaf morphology, Tetraploidy, Somatic embryogenesis

## Abstract

**Background:**

The diploid (2n = 2x = 14) strawberry model plant *Fragaria vesca* ssp. *vesca* ‘Hawaii 4’ was employed for functional analysis of expressed DNA sequences initially identified as being unique to *Fragaria* and of unknown or poorly understood function. ‘Hawaii 4’ is prominent in strawberry research due to its ease of *Agrobacterium*-mediated transformation and regenerability, and its status as the source of the first complete strawberry genomic sequence. Our studies of a set of transformants have documented intriguing, construct-associated effects on leaf morphology, and provide important and unexpected insights into the performance of the ‘Hawaii 4’ transformation and regeneration system.

**Results:**

Following *Agrobacterium*-mediated transformation of leaf explants with gene constructs carried by Gateway® vectors, plants were regenerated using a modified version of an established ‘Hawaii 4’ protocol. Expanding upon the findings of prior studies, we documented that plantlet regeneration was occurring via a somatic embryogenic rather than an organogenic developmental pathway. Among transformants, several variations in leaf morphology were observed. Unexpectedly, a particular leaf variant type, occurring in ~17% of all regenerants independent of construct type, was found to be attributable to tetraploidy. The tetraploidy-associated alteration in leaf morphology could be differentiated from the leaf morphology of diploid regenerants on the basis of a quantitative ratio of leaf dimensions: B/A, where B is the width of the central leaflet and A is the overall width of the trifoliate leaf. Variant effects on leaf morphology of four different transgenic constructs were also documented, and were in all cases distinguishable from the effects of tetraploidy.

**Conclusions:**

These results define opportunities to optimize the existing ‘Hawaii 4’ protocol by focusing on treatments that specifically promote somatic embryogenesis. The reported morphological metric and descriptions will guide future transgenic studies using the ‘Hawaii 4’ model system by alerting researchers to the potential occurrence of polyploid regenerants, and to differentiating the effects on leaf morphology due to polyploidy versus transgenic manipulations. Finally, an intriguing spectrum of leaf morphology alterations resulting from manipulation of expressed sequences of uncertain function is documented, providing a foundation for detailed studies of the respective genes and their functional roles.

## Background

The diploid (2n = 2x = 14) strawberry species *Fragaria vesca* has been embraced as a convenient model system for genomic research. Its favorable attributes include a ~240 Mb reference genome, short generation time, small plant size, close kinship to commercial octoploid strawberry *Fragaria* × *ananassa*, and membership in the economically important Rosaceae family [1,2]. Molecular, genomic, and biotechnological research in strawberry has made substantial progress with respect to in vitro transformation systems [3-5], genetic linkage maps [6-8], transcript analysis [9-11], and genomic sequencing [12-14]. In particular, the *F. vesca* variety known as ‘Hawaii 4’ (PI551572 = CFRA 197) has come to prominence due to its ease of *Agrobacterium*-mediated transformation and regenerability [4,15], the generation of an inbred derivative ‘Hawaii 4x4’ (Slovin J., unpublished), and choice of the ‘Hawaii 4x4’ line as the source of the first complete strawberry genomic sequence [14]. As such, genetic protocol refinement for ‘Hawaii 4’ is an important priority.

The original PI551572 germplasm accession was collected in Hawaii in 1983 by R. Bringhurst, and was maintained by the National Clonal Germplasm Repository (NCGR), Corvallis, Oregon, under the local identification number CFRA 197. *Agrobacterium*-mediated transformation and regeneration was first reported by Haymes and Davis [15]. Those authors described a single transformant, selected on kanamycin and carrying neomycin phosphotransferase (*nptII*) and β-glucuronidase (*gus*) marker genes, and the segregation of these marker genes in a first (R1) generation progeny population. PI551572 had been utilized in this initial effort in part because of two favorable phenotypic traits: it was a so-called ‘Alpine’ or *semperflorens* form, meaning that it had a perpetual (day-neutral) flowering habit as contrasted to the seasonal (short day) flowering habit of most wild strawberry genotypes; and it was a runnering form, in contrast to non-runnering ‘Alpine’ varieties such as ‘Yellow Wonder’ (PI 551827), ‘Baron Solemacher’ (PI 551507), ‘Reugen’ (PI 551834), and ‘Alexandria’ (PI 602923). *F. vesca* is typified by red fruit; however, ‘Hawaii 4’, and ‘Yellow Wonder’ have yellow fruit.

Subsequently, PI551572 was found to be the most favorably responsive among fourteen *F. vesca* germplasm accessions evaluated for transformability and regenerability by Oosumi et al. [4], who established an optimized system that employed hygromycin resistance as the selectable marker. The study of Oosumi et al. [4] was aimed at establishing an in vitro system that would facilitate high throughput development of T-DNA insertion lines [16] in diploid strawberry. Our aim [17-19] and approach differed in that we sought to introduce RNAi constructs, and that our vector system [20] employed kanamycin resistance. As a prelude to future research, we also sought to know whether regeneration was occurring via a somatic embryogenesis or an organogenic pathway, a distinction that had not been specified by Oosumi et al. [4].

Here we report significant new findings regarding the response of ‘Hawaii 4’ to in vitro transformation and regeneration, including explicit evidence that regeneration under the employed conditions occurs via a somatic embryogenic pathway, and the first report that a substantial proportion of the primary transformants of this diploid line are tetraploid. We also report several variant leaf morphologies that are transgene-specific, and describe a simple means of differentiating the altered leaf morphology resulting from tetraploidy from those of the wild type and transgene-related variant forms.

## Results

### Selection and GFP screening of plant transformants

**Figure 1:**
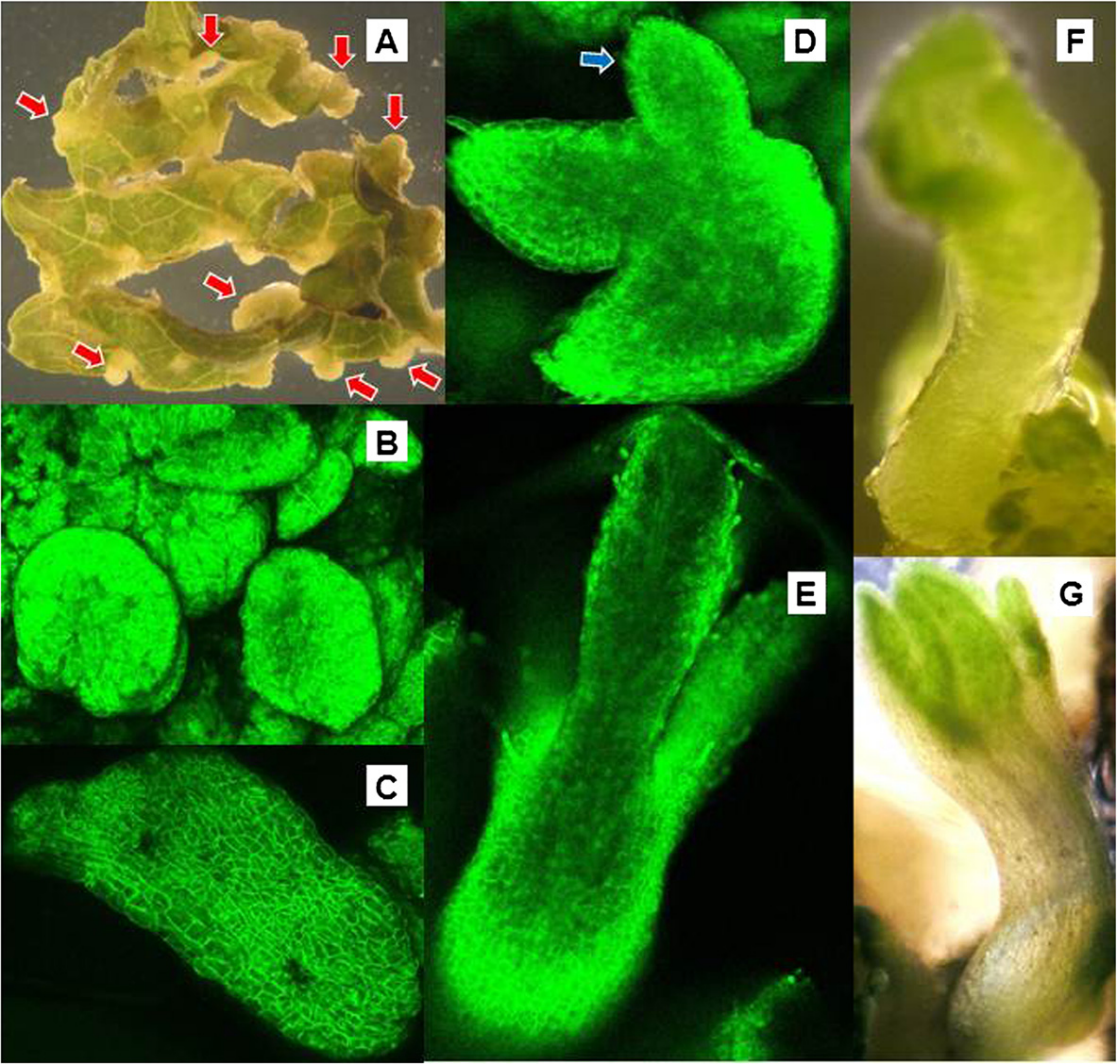
**Stages of somatic embryogenic development.** Photographs in 1A, 1F and 1G were taken on a dissecting microscope, while those in 1B through 1E depict GFP fluorescence as seen under UV illumination via confocal microscopy. **A**. Globular embryonic nodules (red arrows) forming along cut edges of leaf explants. **B**. A cluster of globular embryos on a leaf explant surface. **C**. An early torpedo stage embryo. **D**. Secondary embryo (blue arrow) emerging from a primary embryo. **E**. An advanced stage, bipolar embryo with cotyledonary development. **F**. Bipolar embryo attached to explant at base (root end). **G**. Advanced, bipolar embryos with leaf development and expanding hypocotyl.

Prior to initiating transformation procedures, preliminary experiments were conducted to examine the effect of explant types, combinations of plant growth regulators, antibiotic concentrations, and culture conditions. The results showed that the explants from leaves and petioles responded best on the basal medium with either 3 mg/l BA and 0.2 mg/l IBA, or 2 mg/l TDZ and 0.2 mg/l NAA, on which basis BA and IBA were chosen for subsequent use as the cytokinin and auxin media components. The explants responded more slowly in darkness than under light in the first two weeks of culture. Nodular embryos formed at cut edges of explants after two weeks of culture (Figure 1A). Shoots regenerated after four more weeks in culture. Inclusion of carbenicillin (CB) at 500 mg/l had no observed effect on embryo or callus formation. Inclusion of kanamycin (50 mg/l) caused death of most explants by 5–6 weeks in culture. On the basis of these results, 500 mg/l CB (for suppression of *Agrobacterium* growth) and 50 mg/l kanamycin for transformant selection were included with 3 mg/l BA and 0.2 mg/l IBA in the selection medium. GFP signals were seen on explants after 18 days of co-cultivation. By the third subculture on selective medium, most of the GFP^-^ (and presumably untransformed) cell masses and embryogenic structures on the explants had died. Of the surviving cell masses and embryonic structures, most but not all were GFP^+^.

### Embryogenesis and plant regeneration

Globular somatic embryos formed along some cut edges of the leaf explants during the first week of culture on non-selective medium, and then appeared along all cut edges (Figure 1A - arrows) after transfer of explants to selective medium and culture under light. Isolated single embryos or embryo masses were also produced on explant surfaces. After 4–6 weeks post-inoculation, most of the explants had formed somatic embryo clusters on their cut edges or surfaces (Figure 1B). The explants with GFP^+^ embryo clusters were transferred to non-selective medium for further development. Buds and young shoots grew well on medium with 1–3 mg/l BA and 0.1-0.2 mg/l IBA. GFP screening and microscope observation confirmed that somatic embryos went through heart, torpedo, and dicotyledon stage and developed into whole plantlets (Figure 1C-G). Secondary somatic embryos were formed on some primary dicotyledon stage embryos (Figure 1D - arrow). In order to prevent more secondary regeneration, young shoots and small plantlets were subcultured onto hormone-free medium. Over 1500 well-developed GFP^+^ plants were obtained, and about 1000 plants were transferred to soil and grown to the flowering/fruiting stage. In the exceptional case, the construct FLNH-C08 yielded GFP-positive callus that eventually died in culture even if transferred to kanamycin-free medium, and did not yield any somatic embryos or regenerants in four experiments with 468 inoculated leaf explants.

### Morphological variation among regenerated transformants

**Figure 2:**
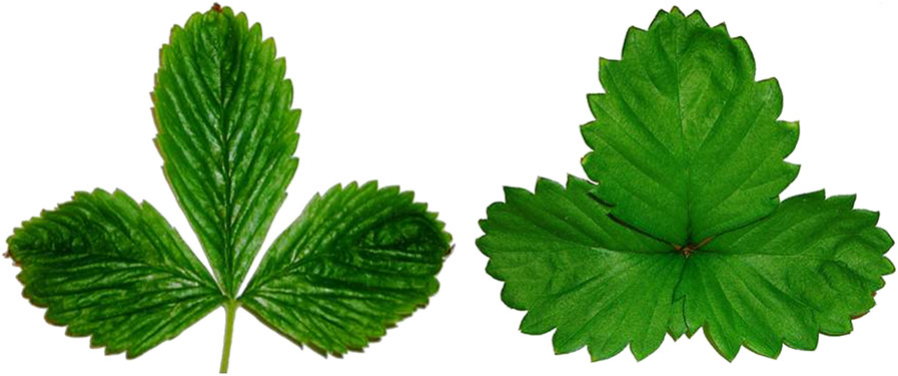
Distinctive, broad leaflet form (right) of a tetraploid regenerant as compared with the normal leaflet form (left) of a diploid regenerant.

By the time plants had reached the three-true-leaf stage of plantlet development, considerable morphological variation was evident among transformants, both within and between constructs. Unexpectedly, a particular broad-leaflet variant form (Figure 2 - right) appeared among the transformants recovered from every construct in which regeneration was obtained. Upon sexual maturity, these broad-leaflet plants displayed enlarged flowers and negligible fruit set.

### Variation due to polyploidy

**Figure 3:**
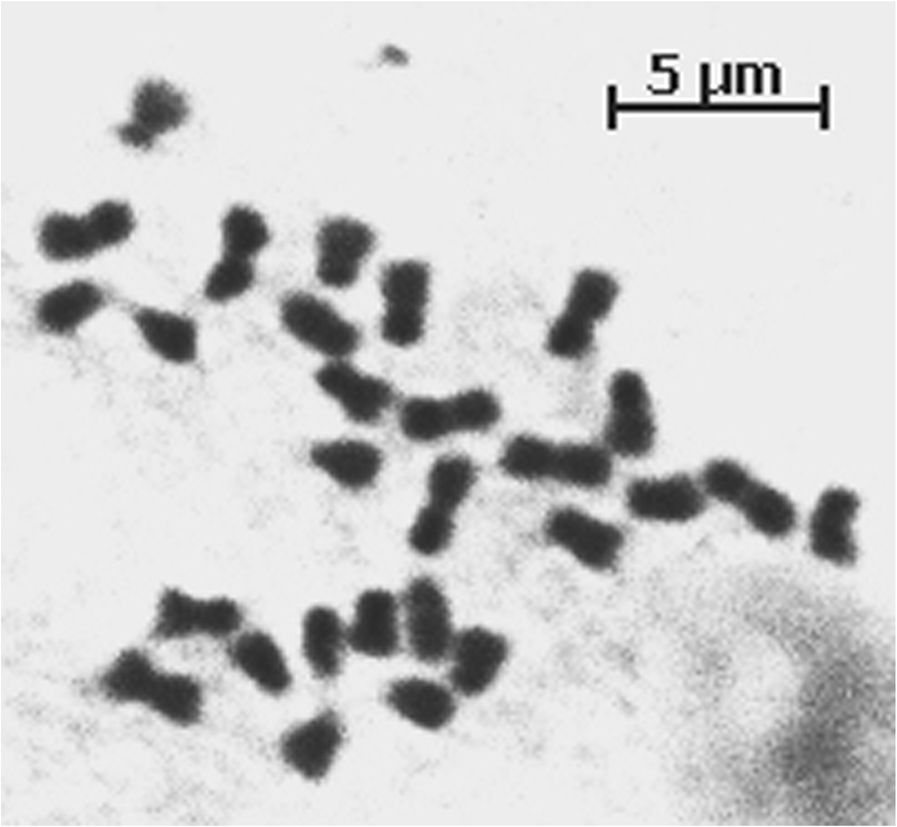
Mitotic chromosome spread displaying 28 chromosomes in root tip cell of a tetraploid regenerant.

Based upon our prior familiarity with colchicine-induced autotetraploidy and its consequences in *F. vesca*[21], it was hypothesized that the broad-leaflet regenerant plants were autotetraploid. A test of this hypothesis, employing nuclear DNA content measurements expressed as proportions of the known standard, indicated that the putative tetraploid plants had DNA content values that were twice those of known diploid comparators. Among a representative sampling of 47 putatively diploid and tetraploid transformant plants, DNA content values ranged from 0.45 to 0.48 for putative tetraploids, and from 0.23 to 0.25 for putative diploids (Table 1). A root tip chromosome count of one plant with an elevated nuclear DNA content showed that it was indeed tetraploid (2n = 4x = 28) (Figure 3).

**Table 1:** **B/A ratios, nuclear DNA content values, of transgenic *F. vesca *** **‘Hawaii 4’ plants**

**Name of plant line**	**B/A ratio**	**Inferred ploidy**	**DNA ratio with internal standard**	**Name of plant line**	**B/A ratio**	**Inferred ploidy**	**DNA ratio with internal standard**
D09-66B	3.8	2x	0.24	E08-64	2.4	4x	0.48
E08-52	3.5	2x	0.24	B07-1	2.3	4x	0.46
D02-30	3.4	2x	0.23	F10-40	2.3	4x	0.46
E08-30	3.3	2x	0.25	E10-56	2.3	4x	0.46
E10-44	3.1	2x	0.23	E11-16	2.3	4x	0.48
B10-6	3.1	2x	0.23	E08-4	2.3	4x	0.48
E08-51	3.1	2x	0.24	E08-16	2.3	4x	0.46
G10-6	3.0	2x	0.23	F06-7	2.2	4x	0.45
F06-13	2.9	2x	0.24	F06-60	2.2	4x	0.48
D02-7	2.9	2x	0.23	D09-28	2.2	4x	0.48
C12-5	2.9	2x	0.23	D09-53	2.2	4x	0.48
F06-174	2.8	2x	0.23	D09-87	2.2	4x	0.48
C10-67	2.8	2x	0.23	E10-53	2.1	4x	0.47
B10-3	2.8	2x	0.25	G10-2	2.1	4x	0.46
E08-1	2.7	2x	0.23	D02-35	2.1	4x	0.48
E08-37	2.7	2x	0.23	C12-9	2.1	4x	0.47
F10-113	2.6	2x	0.23	F10-10	2.0	4x	0.46
D09-14	2.6	2x	0.24	E10-1	2.0	4x	0.45
B07-56	2.5	2x	0.23	G10-131	2.0	4x	0.46
F06-121	2.5	2x	0.23	C10-241	2.0	4x	0.46
**Mean**	**2.95**		**0.23**	D02-25	2.0	4x	0.47
				B10-20	2.0	4x	0.47
				C12-2	2.0	4x	0.47
				B07-12	1.9	4x	0.48
				C10-56	1.9	4x	0.46
				A05-7	1.8	4x	0.47
				F10-42	1.8	4x	0.45
				**Mean**	**2.11**		**0.47**

The internal standard used in DNA content measurements was *Ilex crenata* ‘Fastigiata’.

**Figure 4:**
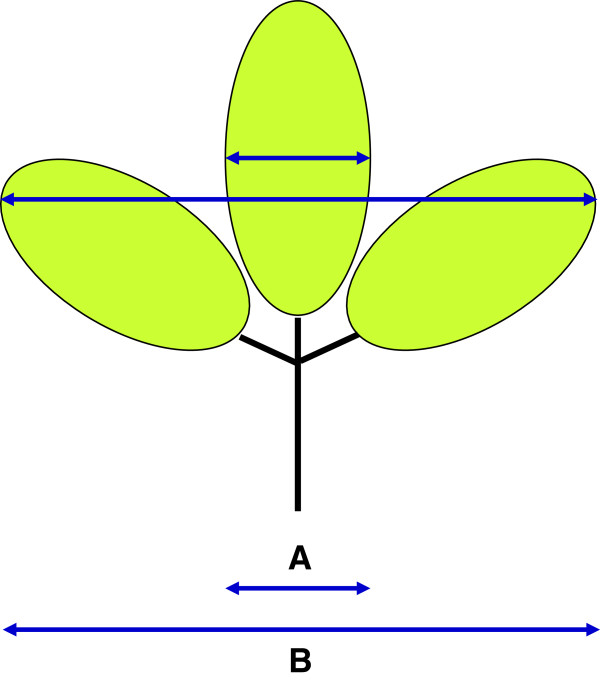
**Depiction of the measured leaf dimensions A and B used in the morphometric assay.** Images of fully expanded trifoliate leaves were measured along the A and B axes as indicated, and the ratio B/A was computed for each leaf.

In comparisons among the 47 plants that were first established by nuclear DNA testing to be diploid or tetraploid, B/A ratios (Figure 4) were determined and found to vary from 2.5 to 3.8 for the diploids, and from 1.8 to 2.4 for the tetraploids (Table 1). The diploid and tetraploid means of 2.95 and 2.11, respectively, differed highly significantly (p <0.01). The lower B/A ratio characteristic of tetraploids resulted from the increased width of the central leaflet (dimension A) relative to the overall width of the trifoliate leaf (dimension B) (Figure 4). Of 992 transformants representing all sixteen constructs, 173 plants, or about 17% of the total, were classified as tetraploid on the basis of their B/A ratios.

### Construct-specific leaf variants

**Figure 5:**
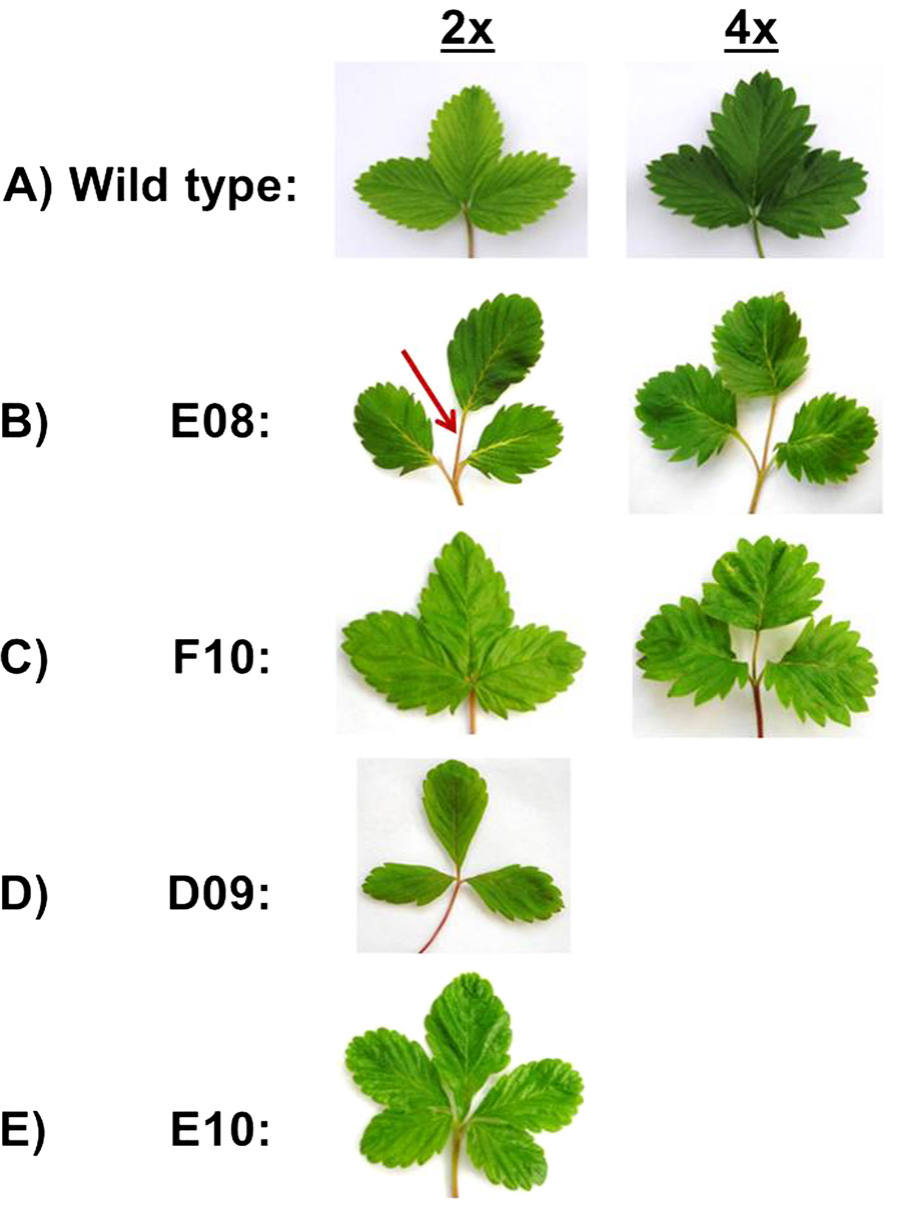
**Variant leaf morphologies in diploid (2x – left) and tetraploid (4x – right) wild type (A), and transformants (B-E) carrying various constructs. A**. Diploid (left) and tetraploid (right) forms of ‘Hawaii 4’. **B**. Construct E08 2x and 4x transformants displaying elongated petiolules (arrow) and subtle alterations in leaflet shape. Leaflets of tetraploid E08 regenerants were broadened as associated with tetraploidy. **C**. The leaves of construct F10 transformants have a ruffled appearance, which is magnified in the 4x form. Elongated petiolules are also sometimes present in the 4x form. **D**. In construct D09 2x transformants, leaves are smaller than wild type, primarily due to narrowing of the leaflets. **E**. In construct E10 transformants, there is a variable tendency to develop leaves with four or five leaflets (see also Figure 6).

With four constructs (FLNH numbers E08, D09, F10, and E10), variant leaf morphologies were observed that were construct-specific, clearly different from wild type (Figure 5A), and clearly distinct from the form associated with tetraploidy. Among the GFP-positive regenerants obtained with these four constructs, only a few (< 10%) displayed construct-specific variant forms,; however, for three of these four constructs (E08, D09, F10), multiple independent transformants that displayed the respective variant phenotypes were obtained, indicating that these features were due to the effects of introducing the construct itself rather than arising from insertional mutation. For the fourth construct, E10, only a single variant transformant was obtained.

### Construct E08

A distinctive suite of morphological effects was generally consistent among six independent transformants obtained with construct E08 (Figure 5B), one of which was tetraploid. Perhaps most obvious among these aberrant features is petiolule length, (petiolule = stalk connecting leaflet to petiole in a compound leaf), which is distinctly elongated in both diploid and tetraploid E08 transformants while inconspicuous in wild type ‘Hawaii 4’ (Figure 5A). The E08 variant plants rarely produce runners, while wild type ‘Hawaii 4’ produces many runners. The variant leaf form associated with construct E08 was stable through vegetative propagation, and was transmitted to a proportion of progeny derived from self-pollination.

### Construct F10

In the case of construct F10, five independent transformants – four diploids and one tetraploid – displayed a subtle, variant leaf form (Figure 5C). The diploids had thinner leaf laminas, lighter color, and markedly shorter petiolules as compared with wild type ‘Hawaii 4’. In the tetraploid, leaflets were wider than in the respective diploids and had a noticeably “ruffled” or corrugated look (Figure 5C).

### Construct D09

Three independent diploid transformants were obtained, and these shared multiple morphological distinctions (Figure 5D) that were stable through vegetative propagation. As compared with ‘Hawaii 4’, these lines had reduced vigor; smaller, narrower leaves; small fruits of ovoid shaped, very few runners, and greatly reduced seed set. Notably, most of the seeds that were set by these lines did not display GFP-fluorescence, and the suite of mutant characters was not transmitted to progeny plants. The recovery of three independent transformants sharing the same suite of features suggests that these features may be due to the effects of the construct itself, rather than arising from insertional mutation.

### Construct E10

**Figure 6:**
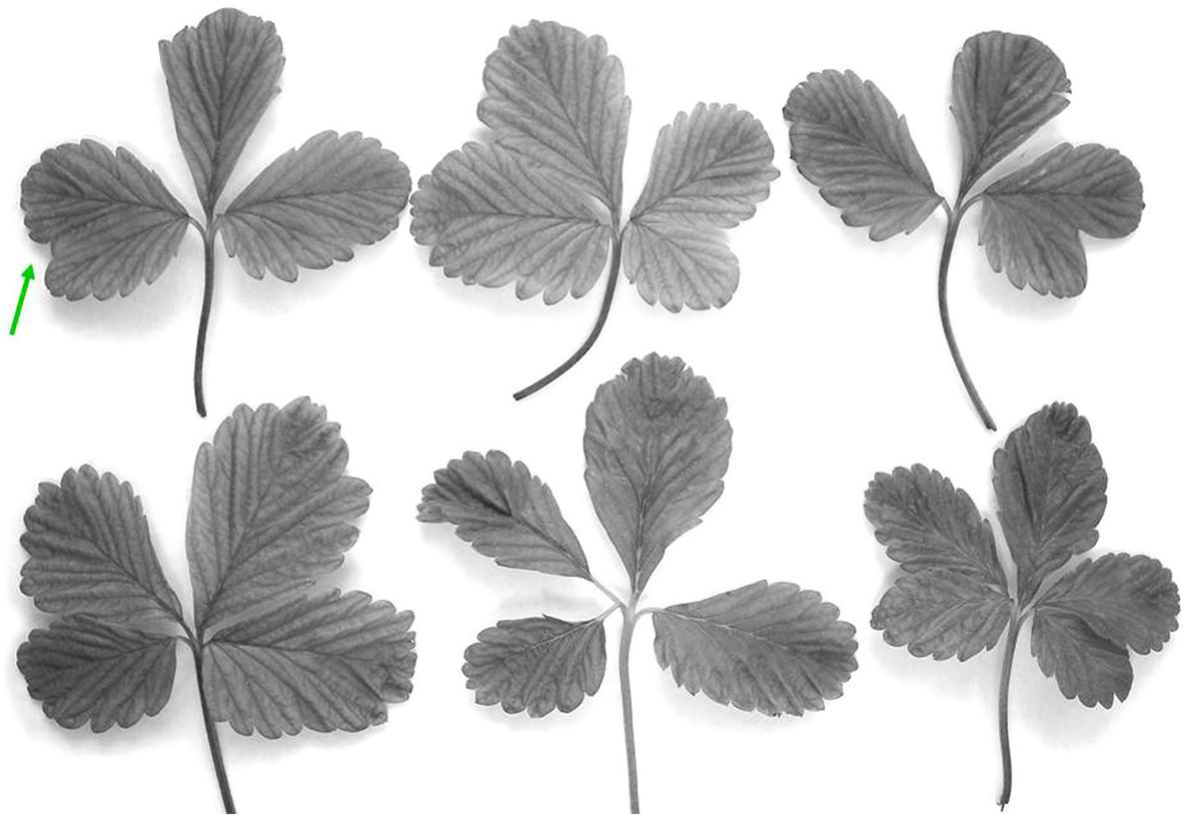
**Variable leaf phenotypes associated with construct E10.** Note presence of four or five leaflets, with instances of partial fusion between adjacent leaflets (arrow).

A noteworthy phenotype consisting of variably increased leaflet number was associated with construct E10 (Figures 5E, 6). This phenotype appeared to varying degrees in three mature (flowering) transformant plants thought to have arisen from a single transformation event, and the variant phenotype was also transmitted to vegetatively propagated runner plants. Although all such plants had mostly trifoliate leaves, some leaves also had four, five, or even seven leaflets, and when a leaf had more than three leaflets sometimes two adjacent leaflets were partially fused (Figure 6).

## Discussion

Our detailed observations of the early stages of transgenic plant regeneration under the employed conditions clearly reveal a pathway of somatic embryogenesis, encompassing heart, torpedo, and dicotyledon stages. This finding contrasts with that of Oosumi et al., [4], who achieved transformation and regeneration of *F. vesca* ‘Hawaii 4’, but who described a regeneration sequence beginning with organogenic shoot formation and followed by rooting upon transfer to hormone-free medium. In one of their figures, the latter authors indicated the presence of “embryonic callus” [4: legend of Figure three], but did not employ the term “somatic embryogenesis” or explicitly specify the nature of their regeneration pathway in their report.

Somatic embryogenesis has been reported in the octoploid, cultivated strawberry [22-26]. Importantly, Husaini and Abdin [24] found that the regeneration pathway from leaflet explants in cultivar ‘Chandler’ was steered toward either somatic embryogenesis or direct shoot formation depending upon the concentration of just one key medium component: thidiazuron (TDZ). This research group then reported the optimization of TDZ concentration for the promotion of somatic embryogenesis in ‘Chandler’ [25], stating that this was the preferred regeneration pathway for their applied research purposes. Other factors found to promote embryogenesis in strawberry include a period of culture in the dark [22,27], and cold treatment [27].

Future efforts toward optimization of post-transformation plantlet regeneration in the widely used ‘Hawaii 4’ variety of the diploid model species *F. vesca* will likely benefit from a targeted approach that seeks to optimize either an organogenic or a somatic embryogenic pathway, depending upon which pathway best serves the project needs. Under the conditions employed in the present study, we documented regeneration via the latter pathway, thereby establishing a defined baseline for future methodological enhancement of the somatic embryogenic approach. Obvious directions for such efforts would be to evaluate the dose-responsiveness of ‘Hawaii 4’ explants to TDZ in comparison to BA, and the effects of culture in darkness or under varying light regimes.

The frequent (~17%) occurrence of tetraploids among post-transformation regenerants was an unexpected outcome. Although numerous reports of *Fragaria* regeneration have appeared, none has yet reported ploidy changes. In our study, the occurrence of tetraploid transformants was not specific to any particular construct sequence, as tetraploids occurred among the transformants that yielded regenerants regardless of construct type. The detection of tetraploidy was an unanticipated, ad hoc observation, for which reason we did not examine the potentially causal affects of experimental factors such as vector system or cultural conditions. Therefore, we cannot separate the various aspects of the transformation and regeneration procedures, considered alone or in concert, as possible causal factors in the induction of tetraploidy based upon available data. However, several intriguing questions are suggested, as discussed below.

First, is ‘Hawaii 4’ particularly susceptible to the induction of tetraploidy, or is such susceptibility a generalized phenomenon in *F. vesca*, in diploid *Fragaria*, or in *Fragaria* in general? The fact that elevated ploidies following in vitro manipulations have not been reported previously in *Fragaria* suggests the possibility that the phenomenon we documented in ‘Hawaii 4’ may have at least an element of genotype- and/or taxon-specificity. Alternately or additionally, the employed vector and delivery system may have been a factor in the tetraploid outcomes. We employed a specific Gateway® vector [20], whereas Oosumi et al., [4] employed the pCAMBIA-1304 binary vector, while our study and theirs both employed an *Agrobacterium*-based delivery system. Oosumi et al. [4] did not report the occurrence of tetraploidy among their transformants; however, a careful examination of the four transformants that were photographically documented by these authors suggests to us that the transformant depicted in their Figure eight-b [4] may be a tetraploid, as suggested by the distinctive leaf morphology that we have shown to be indicative of tetraploidy.

The tetraploid transformants of ‘Hawaii 4’ that we examined all shared a distinctive leaf morphology, which manifested as a quantifiably altered ratio (B/A) of central leaflet to overall leaf width. Increased cell size is a widely documented and general consequence of ploidy elevation, at least from the diploid to the tetraploid level. It is possible that increased cell size alone may account for the disproportionate broadening (versus lengthening) of all three leaflets of the strawberry trifoliate leaf, the net result of which is that the broadening of the central leaflet is proportionately greater than the overall broadening of the trifoliate leaf. As yet we have not defined the tetraploidy-associated change in leaf morphology at the cellular level. We have, however, thoroughly documented the fact of tetraploidy, and have provided a simple morphological metric that allows its detection and distinction from diploid plants among regenerant transformants of ‘Hawaii 4’.

The recognition that tetraploidy occurs frequently and that it has a distinctive phenotype when it does occur in ‘Hawaii 4’ transformants will enhance the ability of researchers to identify mutant, non-tetraploid forms in mutagen-treated and/or transformed ‘Hawaii 4’ plants. In the present study, we obtained transformants with variously altered leaf morphologies, and on the basis of our characterization of the effects of tetraploidy were able to partition ploidy-related from construct-associated alterations in leaf morphology, even when both occurred in the same plant. Thus, the petiolule elongation associated with introduction of construct E08 was present in both diploid and tetraploid regenerants (Figure 5B), and the tetraploid form was clearly distinguishable by its widened leaflets (Figure 5B - right) and altered B/A ratio. Contrastingly, the ruffled leaflet phenotype associated with construct F10 was considerably magnified in the tetraploid, as compared with the diploid, form (Figure 5C).

A useful catalogue of morphological features was provided by Slovin et al. [28], pertaining to *F. vesca* inbred line 5AF7, a yellow-fruited and runnerless ‘Alpine’ genotype closely related to ‘Hawaii 4’, which itself is a runnering variety. This comprehensive phenotypic description was intended to provide a baseline to which other forms including derivative mutant forms could be compared. Petiole length was among the features described; however, no mention was made of petiolule length. The petiolule elongation displayed by E08 transformants reveals and provides definition to an additional variable trait to be found in *Fragaria*, thus adding to the useful trait catalogue contributed by Slovin et al. [28].

Another interesting leaf phenotype occurred in association with construct D09 (Figure 5D), and this phenotype was represented only in diploid regenerants. Here, the leaves were of slightly reduced size, mostly due to narrowing of the leaflets, giving the plant an overall gangly look. Finally, several regenerants carrying construct E10 exhibited a variable increase in leaflet number, from the usual three to four, five, or even seven, wherein adjacent leaflets were sometimes partially fused (Figures 5E, 6). Pentafoliate leaves are a defining feature of diploid *Fragaria* species *F. pentaphylla*, a form indigenous to the Tibetan region. However, in *F. pentaphylla*, the additional leaflets are quite small or vestigial, and are attached much lower on the petiole [29], while in E10 the additional leaflets are attached at more-or-less the same point as the normal three leaflets (Figure 6). The instability of the E10 variant form and its occurrence in only one confirmed independent transformant invokes the possibility of an insertional or other mutagenic cause. A somaclonal variant of cultivated strawberry (*Fragaria × ananassa*) variety ‘Redcoat’ with a similar phenotype was described by Nehra et al. [30].

## Conclusions

The results reported here define opportunities to optimize the existing ‘Hawaii 4’ protocol by focusing on treatments that specifically promote somatic embryogenesis. The reported morphological metrics and descriptions will guide future transgenic studies using the ‘Hawaii 4’ model system by alerting researchers to the potential occurrence of polyploid regenerants, and to differentiating the effects on leaf morphology due to polyploidy versus transgenic manipulations. The results reported here raise many intriguing questions about the transformation system and host genotype employed in the present study. Was tetraploidy induced by some aspect of the regeneration system, or did the polyploids arise from polyploid cells pre-existing in the explant material? To what extent was the host genotype a contributing factor? For constructs associated with altered phenotypes, why did only a small proportion of regenerated, GFP + plants display the associated aberrant phenotype? Yet aside from the generation of biological and methodological puzzles that invite further study, the present investigation also yielded intriguing mutant lines displaying a remarkable spectrum of leaf morphology alterations and resulting from manipulations of previously uncharacterized genes, thereby opening new avenues to the characterization of novel gene functions.

## Methods

### Vector construction and manipulation

Expressed sequences of unknown function were identified in partial sequences from 5′ end sequencing of fruit and flower cDNA libraries. Sequences were characterized as “unknown” if BLAST analysis revealed an expectation (E) value <10^-3^. Preference was given to sequences lacking domain structure, as determined by tests against INTERPRO (http://www.ebi.ac.uk/interpro/) databases. As a prelude to future functional analyses using RNAi suppression, constructs containing the sequences of interest were introduced into *F. vesca* ‘Hawaii 4’ using Agrobacterium-mediated transformation, as described below.

The Gateway® vector system (Invitrogen, Carlsbad, California) [20] was employed. Entry clones (in donor vector, pDONR™222) with constructs of sixteen “unknown” gene fragments in the range of 700–1800 bp were developed using standard procedures. Constructs were mobilized to the RNAi destination vector, [pK7GWIWG2D (II), 0] [20] by LR reaction according to the manufacture’s instruction (Gateway® LR Clonase™ II enzyme, Invitrogen). To verify LR product sizes/identities for each construct, the plasmids were isolated from 4 to 8 clones and digested with restriction enzyme *Bsr*GI and analyzed on agarose gels.

The expression clones were transformed into *Agrobacterium* strain GV3101 by electroporation. *Agrobacterium* colonies were verified by colony PCR using GFP marker gene primers GFP-F1 (5′-CGGCGGCGGTCACGAACTC) and GFP-R1 (5′-CACCTACGGCAAGCTGACCCTGAA); and construct-specific primers (not shown).

### Plant in vitro culture media

MB5 medium (MS salts, [31] with B5 vitamins [32] was used as basal medium, with 0.8% (w/v) agar in culture plates and vessels. Plant growth regulators, vitamins and antibiotic solutions were filter-sterilized and added into the basal medium post-autoclaving, when the medium temperature had dropped to about 50°C.

### Plant material for transformation

*Fragaria vesca* ‘Hawaii 4’ seeds were sterilized in 30% (v/v) commercial bleach (6% NaOCl) solution with 2 drops Tween 20® for 10 minutes followed by rinsing them four times with sterile distilled water, and then inoculated onto agar plates of MB5 at pH 5.8 with 3% (w/v) sucrose. Germinating seeds were cultured initially at 22°C under dim light (8 μmol m^-2^ s^-1^) for two weeks, then at 25°C under 12 hours photoperiod (light intensity 124 μmol m^-2^ s^-1^). Seedlings with one or more true leaves were transferred into Magenta GA7® boxes containing basal medium to attain increased size. The seedlings were routinely transferred into fresh medium two weeks prior to their use as explant sources.

### Explant co-cultivation

The co-cultivation medium (coMB5) was MB5 at pH 5.5, with 2% (w/v) sucrose, to which other components were added only as specified below. Young, fully expanded leaflets were placed adaxial side up on two layers of sterile filter paper wetted with sterile water in a Petri dish and sliced with a scalpel to produce multiple cuts across and/or along the secondary veins, thereby producing an abundance of wound sites while keeping the overall structure of the leaflet intact as a unit to enable ease of handling. The three leaflets of each leaf were also kept connected until and during the incubation with *Agrobacterium*, but were separated by cutting after incubation or (best) after washing off the *Agrobacterium*. Freshly prepared explants were used directly for co-cultivation with *Agrobacterium*, then plated onto selective medium as described below, with 20 leaf explants per plate. About 50–100 leaflets were used as explants for each construct.

*Agrobacterium* with specific constructs were cultured in 3 ml LB medium with 30 mg/l rifampicin, 50 mg/l gentamycin and 100 mg/l spectinomycin at 28°C in a shaker for about 20–22.5 hours (OD600, 1.1-2.8). The bacteria were washed with liquid coMB5 medium and then resuspended in 25 ml coMB5 medium with 10 ul 0.25 μM acetosyringone. The final OD600 of the cells used for co-cultivation was 0.15-0.34.

For co-cultivation, the freshly sliced explants were placed into the *Agrobacterium* suspension and incubated on a shaker (50–90 rpm) for 25–40 minutes at room temperature. The explants were then quickly blotted dry on sterilized filter paper and then placed abaxial side up in 0.8% (w/v) agar culture plates of coMB5 plus 3 mg/l 6-benzylaminopurine (6-BA) and 0.2 mg/l indole-3-butyric acid (IBA). After co-culture for 2–3 days at room temperature in the dark, the explants were rinsed 2–3 times with 15–30 ml liquid coMB5 medium with 500 mg/l carbenicillin (CB), then blotted dry on sterilized filter paper, then cut into smaller pieces and put on MB5 medium at pH 5.8 with 2% (w/v) sucrose with 500 mg/l CB, 3 mg/l 6-BA and 0.2 mg/l IBA to develop for 7–8 days at room temperature in the dark.

### Selection of transformants

The explants, which had expanded in size during culture, were then cut again into smaller pieces before transfer to MB5 selective medium containing 50 mg/l kanamycin and 500 mg/l CB, and subsequent culture under the light. For all subsequent procedures of selection and regeneration, the MB5 medium was at pH 5.8, with 3% (w/v) sucrose and other components as specified below.

The selection plates were cultured under light for 12 hours photoperiod (light intensity 124 umol m^-2^ s^-1^) at 25°C. The explants were first cultured for 8–12 days on MB5 medium with 3 mg/l 6-BA, 0.2 mg/l IBA, 50 mg/l kanamycin and 500 mg/l CB, then subcultured twice at two to three-week intervals on this medium, followed by repeated subculture at a reduced level of 25 mg/l kanamycin and 250 mg/l CB. GFP fluorescence was periodically assayed after five weeks in culture. Subsequently, the GFP positive clusters or individual shoots were transferred to the same medium without kanamycin, but that was supplemented with CB at 100 mg/l if any *Agrobacterium* contamination was evident, and subcultured every 4 weeks. In general, shoots developed into plantlets on agar medium without hormones; however, up to 0.2 mg/l BA was included to stimulate growth when shoot development was not vigorous. Plantlets with roots were transferred into soil after washing off the agar. All explants were screened for GFP expression signals after each subculture on selective medium by viewing plates under UV illumination on a confocal microscope.

### Fluorescence confocal microscopy

Confocal microscopy was performed at the University of New Hampshire Confocal Imaging Center. The selection of GFP positive transformants was done by observation of tissues with a Zeiss LSM 510 Meta Confocal laser scanning microscope equipped with a fluorescein isothiocyanate (FITC) filter.

### Nuclear DNA content measurements

Relative quantification of nuclear DNA contents was provided by Plant Cytometry Services (Schijndel, Netherlands). Young, folded leaves were harvested, placed in labeled microfuge tubes on ice, and shipped via overnight currier to the service provider. In the provided assay, the fluorescent stain, 4′, 6-diamidino-2-phenylindole (DAPI) was used to bind the nuclei and DNA ratios between each sample internal standard and a known standard, *Ilex crenata* Thunb. f. ‘Fastigata’, were determined. Samples from various *Fragaria* species of known ploidy, including 2x and 4x forms of *F. vesca* ‘Baron Solemacher’ [21] were also assayed as comparators.

### Leaf morphology measurements

Based upon initial observation that the leaflets of 4x plants (as confirmed by nuclear DNA content measurement) looked broader than those of 2x plants, a quantitative morphometric assay was devised, as depicted in Figure 2. Measurement was facilitated by generating conveniently preservable, actual-size scanned images of each leaf. The dimensions “A” (width of central leaflet at its widest point) and “B” (transect between tips of the two lateral leaflets) were measured (Figure 1), and the ratio B/A was computed for one representative leaf per plant for 992 transformants. The B/A ratios of initially identified sets of 2x and 4x plants were compared using a Student’s T-test (one-tailed).

### Chromosome count

Root tips were taken from tetraploid transformant F06-75, and aceto-orcein-stained squash preparations were prepared as described by Nathewet et al. [33]. Actively growing root tips were collected and pre-treated in 0.002 M 8-hydroxyquinoline solution at room temperature for 1 hr and subsequently stored at 4°C for 15 hrs). The root tips were then rinsed in 0.075 M KCl briefly and fixed in 3:1 ethanol: acetic acid at 4°C for at least 24 hrs. Fixed root tips were softened in 1 N HCl at 60°C for 20 min, then rinsed briefly in distilled water prior to slide preparation and examination.

## Abbreviations

6-BA: 6-benzylaminopurine; CB: Carbenicillin; GFP: Green fluorescent protein; IBA: Indolebutanoic acid; NAA: 1-naphthaleneacetic acid; TDZ: Thidiazuron; MS medium: Murashige and Skoog medium; MB5: MS salts and B5 vitamins; LB medium: Luria-bertani broth; FLNH: Florida New Hampshire.

## Competing interests

The authors declare that they have no competing interests.

## Authors’ contributions

TMD and KMF designed and managed the research, QZ performed most of the experimental work and data gathering. All three authors contributed to manuscript writing, and approved the final manuscript.
